# ^77^Se-Enriched Selenoglycoside Enables Significant Enhancement in NMR Spectroscopic Monitoring of Glycan–Protein Interactions

**DOI:** 10.3390/pharmaceutics14010201

**Published:** 2022-01-15

**Authors:** István Timári, Sára Balla, Krisztina Fehér, Katalin E. Kövér, László Szilágyi

**Affiliations:** 1 Department of Organic Chemistry, University of Debrecen, Egyetem tér 1, H-4032 Debrecen, Hungary; timari.istvan@science.unideb.hu (I.T.); balla.sara@science.unideb.hu (S.B.); 2 Department of Inorganic and Analytical Chemistry, University of Debrecen, Egyetem tér 1, H-4032 Debrecen, Hungary; feher.krisztina@science.unideb.hu; 3 Molecular Recognition and Interaction Research Group, Hungarian Academy of Sciences, University of Debrecen, Egyetem tér 1, H-4032 Debrecen, Hungary

**Keywords:** ^77^Se isotope, chemical synthesis, glycan, HSQMBC, lectin, ligand–protein binding, NMR spectroscopy, selenodigalactoside, TDG

## Abstract

Detailed investigation of ligand–protein interactions is essential for better understanding of biological processes at the molecular level. Among these binding interactions, the recognition of glycans by lectins is of particular importance in several diseases, such as cancer; therefore, inhibition of glycan-lectin/galectin interactions represents a promising perspective towards developing therapeutics controlling cancer development. The recent introduction of ^77^Se NMR spectroscopy for monitoring the binding of a selenoglycoside to galectins prompted interest to optimize the sensitivity by increasing the ^77^Se content from the natural 7.63% abundance to 99%. Here, we report a convenient synthesis of ^77^Se-enriched selenodigalactoside (SeDG), which is a potent ligand of the medically relevant human galectin-3 protein, and proof of the expected sensitivity gain in 2D ^1^H, ^77^Se correlation NMR experiments. Our work opens perspectives for adding isotopically enriched selenoglycans for rapid monitoring of lectin-binding of selenated as well as non-selenated ligands and for ligand screening in competition experiments.

## 1. Introduction

Mounting awareness in recent decades has highlighted the importance of molecular events in how lectins, a class of carbohydrate-binding proteins, recognize cell surface glycans [1]. Lectin–glycan interactions are involved in cellular processes such as adhesion, intercellular communication, growth and differentiation, cell cycle, and apoptosis. As a result, lectin-mediated processes are implicated in wide range of diseases such as cancer, inflammation and fibrosis, heart disease, and stroke [2,3,4,5,6,7]. Galectins, Gal-1, -3, -7, and -9 in particular, have been implicated in multiple ways in malignant cell proliferation processes [4,5,7,8]. Inhibition of glycan–galectin interactions thus offers a promising perspective towards developing therapeutics influencing these processes. Research on small-molecule- or multivalent glycomimetics as lectin inhibitors is especially worth mentioning in this aspect [9,10,11].

Several analytical techniques (surface plasmon resonance—SPR, isothermal titration calorimetry—ITC, nuclear magnetic resonance—NMR, or X-ray crystallography) are available to investigate these interactions. Among these methods, NMR spectroscopy stands out as it allows studying protein–glycan interactions in solution, i.e., close to physiological conditions at the molecular level [12,13]. Observation of NMR signals of the ligand molecules binding to protein receptors is one of the most powerful approaches to gain insight into structural and dynamic aspects of these interactions [14,15]. NMR spectroscopy is plagued, however, with sensitivity issues, representing a double challenge because of low concentrations and typically weak interactions. Signal overlap is another problem to be dealt with, especially with carbohydrate ligands typically having narrow ^1^H chemical shift ranges with consequent serious overlaps in the spectra [16]. Spectral overcrowding quickly becomes unmanageable with oligosaccharide ligands or with carbohydrate mixtures, such as in studies of potential competition with different ligands. ^13^C- or ^15^N-based NMR approaches are much less affected by spectral overlap, but suffer even more from low sensitivity at natural abundance of these NMR-active isotopes even in sensitivity-enhanced ^1^H-detected versions. A continuous methodological challenge in NMR-spectroscopical monitoring is to expand the range of techniques and, here, the introduction of distinct NMR sensors beyond the commonly used ^1^H, ^13^C, and ^15^N isotopes comes into play. Application of ^19^F as a reporter nucleus has been proposed as an alternative, combining high sensitivity (0.83% compared with that of ^1^H) with superior selectivity owing to a large chemical shift range, as well as specific labeling used to introduce this label just to a few predefined positions of the molecule of interest (typically substituting OH for F) [17,18,19,20]. It is to be noted, however, that fluorinated derivatives are chemically distinct from their natural counterparts; still, very often, they turn out to be good mimics of the parent molecules in terms of structure and binding properties.

The success of the ^19^F-approach prompted us to look for a further isotope to be introduced as an NMR reporter into lectin–oligosaccharide interaction analysis. Inspired by the discovery that thiodigalactoside (TDG) is a potent ligand for adhesion/growth-regulatory galactose-binding lectins [21,22,23], we have tested selenodigalactoside (SeDG) as a binding partner and its ^77^Se isotope as an NMR-spectroscopical sensor. As we have detected rather equal affinities of TDG and SeDG to human galectins-1 and -3 (Gal-1/-3) [24], we initiated ^77^Se NMR-based monitoring of SeDG binding to these lectins [25,26]. ^77^Se is a spin-½ NMR isotope with broad chemical shift range (ca. 3000 ppm), which makes it promising as a selective probe [25,27,28,29]. Direct ^77^Se NMR detection [30,31] is, however, unfavorable owing to the low natural abundance (7.63%) and decreased sensitivity (0.7 of ^1^H) of this nucleus. To improve sensitivity, we proposed a 2D ^1^H-^77^Se HSQMBC (heteronuclear single quantum multiple-bond correlation) experiment via indirect ^77^Se detection using CPMG-INEPT (Carr–Purcell–Meiboom–Gill insensitive nuclei enhanced by polarization transfer) out-and-back ^1^H→^77^Se→^1^H polarization transfer. Theoretically, this approach can yield up to 60-fold ((γ_H_/γ_Se_)^2.5^) sensitivity enhancement, but, owing to competing relaxation and other transfer processes, a sensitivity gain about 20 can be realized in practice [25,28,32].

A next step toward optimizing ^77^Se sensor capacity is to prepare the lectin ligand as an isotopically enriched probe, that is, bringing isotope representation from the natural 7.63% abundance to close to 100%. To exploit this attractive potential, we provide an example by the synthesis of a sample of di(β-D-galactopyranosyl)selenide enriched to 99% in ^77^Se isotope ([^77^Se]DGal). Exploratory experiments reported here with this ^77^Se-enriched material indeed confirmed our expectations for a spectacular increase in NMR detection sensitivity. We will furthermore demonstrate that combining our advanced ^1^H-^77^Se HSQMBC NMR method with ^77^Se enrichment enables rapid monitoring of lectin-binding of selenated as well as non-selenated ligands and high-throughput screening in competition experiments.

## 2. Materials and Methods

### 2.1. Synthetic Procedures

#### 2.1.1. ^77^Se-Enriched Di(2,3,4,6-tetra-*O*-acetyl-β-D-galactopyranosyl)selenide (2)

An ethanolic solution of NaH^77^Se was prepared following a procedure described by Klayman and Griffin [33]. In a flame-dried flask, isotopically enriched (99%) 77-selenium powder (Laboratory Standards Kft. Budapest, Hungary, lot no. SELM-2445-PK) (50 mg, 0.65 mmol) was stirred in dry ethanol (5 mL) under argon atmosphere (Note). After cooling in an ice bath, sodium borohydride (50 mg, 1.3 mmol) was added and stirring continued until the selenium was dissolved and gas evolution subsided (ca. 5 min). To this clear solution, 1-Br-2,3,4,6-tetra-*O*-acetyl-α-D-galactopyranose (**1**, 520 mg, 1.3 mmol) dissolved in dry methyl cyanide (2 mL) was added and stirring continued at room temperature until TLC (hexane/EtOAc 1:1) indicated consumption of **1** (2 h). The reaction mixture was evaporated under reduced pressure, the syrupy residue dissolved in dichloromethane, it extracted with water twice, the organic phase dried (MgSO_4_), and the solvent was removed in vacuo to yield **2** as a pale-yellow solid at 323 mg (69%). According to its ^1^H NMR spectrum, this product was judged to be sufficiently pure, apart from some residual signals from ethanol (1.23/3.70 ppm, see Figure S1, bottom), to be deacetylated in the next step (2.1.2). [α]_D_^22^—5.2 (c 0.2 CHCl_3_); ^1^H NMR (CDCl_3_, 500 MHz): δ (ppm) 5.46 (br.d, 1H, H-4, *J*_3.4_ 3.0 Hz); 5.30 (t, 1H, H-2, *J*_1,2_ = *J*_2,3_ 10.3 Hz); 5.06 (d, 1H, H-1); 5.05 (br.d, 1H, H-3); 4.17 (dd, 1H, H-6a, *J*_6a,6b_ 11.2 Hz, *J*_5,6a_ 6.7 Hz); 4.12 (dd, 1H, H-6b, *J*_5,6b_ 6.5 Hz); 3.90 (m, 1H, H-5); 1.99; 2.05; 2.06; 2.17 (12H, 4 CH*_3_*(CO)); ^13^C NMR (CDCl_3_, 125 MHz): δ (ppm) 169.7; 169.9; 170.1; 170.3 (4 CH_3_(***C***O)); 77.0 (C-1, *^1^J*_Se,C1_ 88.7 Hz); 75.8 (C-5); 71.6 (C-3, *^3^J*_Se,C3_ 5.4 Hz); 68.1 (C-2, *^2^J*_Se,C2_ 16.2 Hz); 67.2 (C-4); 61.6 (C-6); 20.74, 20.65(2x), 20.74 (4x***C***H_3_(CO)); HRMS: calc. for C_28_H_38_O_18_^77^Se: [M + Na]^+^:762.116, found: 762.115.

#### 2.1.2. ^77^Se-Enriched Di(β-D-galactopyranosyl)selenide (3)

To a solution of ^77^Se-enriched di(2,3,4,6-tetra-*O*-acetyl-β-D-galactopyranosyl)selenide (**2**) (180 mg) in dry methanol (50 mL), a 25% solution of sodium methoxide in methanol (0.06 mL) was added. After 15 min at room temperature, 0.5 g of Amberlist^®^ 15H ion exchange resin was added, the resin filtered off after 5 min of stirring, and the filtrate evaporated to dryness under reduced pressure. Recrystallization from methanol furnished 94 mg of white solid (96%). ^1^H NMR (Figure S2) and ^13^C NMR (Figure S3) spectra of this product (**3**) are provided in the Supplementary Material. [*α*]_D_^22^—30.9 (c 0.2 H_2_O); ^1^H NMR (DMSO-*d_6_*, 500 MHz): δ (ppm) 4.78 (dd, 1H, H-1, *J*_1,2_ 9.8 Hz, *^2^J_H_*_2,Se_ 6.1 Hz); 3.70 (m, 1H, H-4); 3.45–3.53 overlapping signals (3H, H-2, H-6a, H-6b); 3.32 (m, 1H, H-5); 3.28 (m, 1H, H-3); ^13^C NMR (DMSO-*d_6_*, 125 MHz): δ (ppm) 80.4 (C-5); 79.8 (C-1, *^1^J*_Se,C1_ 80.4 Hz); 74.6 (C-3, *^3^J*_Se,C3_ 4.6 Hz); 71.0 (C-2, *^2^J*_Se,C2_ 12.8 Hz); 68.4 (C-4); 60.4 (C-6); HRMS: calc. for C_12_H_22_O_10_^77^Se [M + Na]^+^: 426.030, found: 426.032.

Note: Exclusion of traces of water and of oxygen is important to avoid losses of selenium via formation of hydrogen selenide and/or oxidation. Any hydrogen selenide (which is very poisonous) that might have formed was trapped by passing it into a 5% aqueous solution of lead acetate.

### 2.2. Sample Preparation for NMR Measurements

For ^77^Se-NMR-based monitoring of ligand binding to *h*Gal-3, 10 μL of a 120 mM [^77^Se]DGal ligand stock solution (in a 10 mM phosphate buffer in D_2_O, pH 7.4, containing 0.5 M NaCl) and 590 μl 10 mM phosphate buffer in D_2_O, pH 7.4, containing 0.5 M NaCl were pipetted into a 5 mm NMR tube to obtain a 2.0 mM final concentration of [^77^Se]DGal ligand. In a second step, *h*Gal-3 protein was added as lyophilized powder to reach a *h*Gal-3/[^77^Se]DGal ratio of 0.0145:1 (c_hGal-3_ = 29 μM) in the NMR tube. This sample was used for binding studies with the ^1^H-^77^Se CPMG-HSQMBC experiment. Thiodigalactoside (TDG) was added as a solid material (0.43 mg) to the previous sample, resulting in a *h*Gal-3/[^77^Se]DGal/TDG ratio of 0.0145:1:1 (29 μM/2 mM/2 mM) in the NMR sample measured in the third step of the ^1^H-^77^Se CPMG-HSQMBC binding experiments. ^1^H NMR spectra obtained on samples of each step of binding experiments can be seen in Figure S4. It is noteworthy that the protein–ligand concentration ratio in these NMR experiments should always be optimized in accordance with the pertinent binding affinity of the system being studied.

### 2.3. NMR Measurements

^1^H-^77^Se CPMG-HSQMBC NMR experiments were performed on a Bruker Avance I spectrometer (Bruker BioSpin GmbH, Rheinstetten, Germany) operating at 400 MHz ^1^H frequency equipped with a BBI z-gradient probe. All experiments were carried out at 303 K, and NMR data were processed with TopSpin 2.1 or 3.5 (Bruker Biospin GmbH, Karlsruhe, Germany).

^1^H-^77^Se correlation spectra were measured by the refocused 2D ^1^H-^77^Se CPMG-HSQMBC pulse sequence with ^77^Se decoupling [25]. Simultaneous composite π pulses on the ^1^H and ^77^Se channels were applied with an equal duration of 90° pulses (18 μs) achieved with careful adjustment of power levels. The CPMG-INEPT delay Δ for long-range heteronuclear coupling evolution was adjusted to 45.1 ms. For ^77^Se CPD decoupling during FID acquisition (183 ms), the WALTZ16 scheme with a 90° pulse length of 400 μs was used. 2D ^1^H-^77^Se CPMG-HSQMBC spectra were recorded for sensitivity comparison on [^77^Se]DGal and SeDGal samples, with 1024 total data points in the ^1^H (*t*_2_) dimension and 32 total points in the ^77^Se (*t*_1_) dimension, using spectral windows of 6.99 ppm (2796 Hz) for ^1^H and 6.00 ppm (458 Hz) for ^77^Se. On [^77^Se]DGal sample, 8 scans per t_1_ increment were accumulated; in contrast, 360 scans per t_1_ increment were used for SeDGal sample. The polarization recovery delay between consecutive scans, *d1*, was set to 1.7 s. In binding experiments, each 2D ^1^H-^77^Se CPMG-HSQMBC spectrum was recorded with 1024 total data points in the ^1^H (*t*_2_) dimension and 2 total points in the ^77^Se (*t*_1_) dimension, using spectral windows of 6.99 ppm (2796 Hz) for ^1^H and 6.00 ppm (458 Hz) for ^77^Se. 32 scans per t_1_ increment were accumulated and the polarization recovery delay between consecutive scans, *d1*, was set to 1.7 s.

## 3. Results and Discussion

### 3.1. Synthesis of ^77^Se-Enriched Di(β-D-galactopyranosyl)selenide, [^77^Se]DGal

**Scheme 1:**
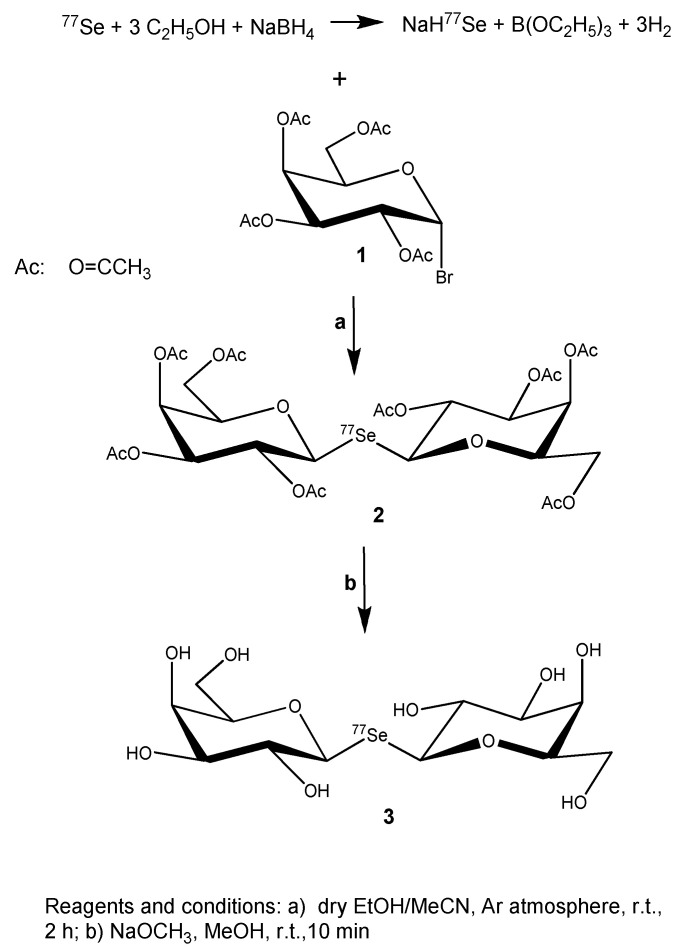
Synthesis of ^77^Se-enriched selenodigalactoside ([^77^Se]DGal, **3**).

Preparation of this compound was based on the reaction of 1-Br-2,3,4,6-tetra-*O*-acetyl-α-D-galactopyranose (**1**) with NaH^77^Se, with the latter obtained by NaBH_4_ reduction of elemental selenium [33] 99% enriched in ^77^Se isotope. Reaction of (**1**) with NaH^77^Se furnished ^77^Se-enriched octa-*O*-acetyl-di(β-D-galactopyranosyl)selenide (**2**), which gave the desired product, ^77^Se-enriched selenodigalactoside ([^77^Se]DGal **3**), upon Zemplén deacetylation (Scheme 1). This two-step synthesis was carried out with a good overall yield; further details of the reactions are given in Section 2.

### 3.2. NMR Experiments

As mentioned in the introduction, we have recently proposed a 2D ^1^H-^77^Se heteronuclear correlation experiment to enhance the detection sensitivity of ^77^Se NMR by replacing direct NMR observation on ^77^Se for indirect observation on ^1^H nuclei [25,26]. Our 2D ^1^H-^77^Se CPMG-HSQMBC pulse sequence is based on out-and-back ^1^H→^77^Se→^1^H polarization transfer via ^2,3^*J*(^1^H,^77^Se) long-range couplings. CPMG-INEPT long-range transfer eliminates signal phase modulation and significantly reduces intensity losses from co-evolving *J*(^1^H, ^1^H) couplings. Furthermore, it can suppress line broadening from chemical exchange, ensuring maximum detection sensitivity and clean signal phases, as was described earlier [25,26,32].

**Figure 1:**
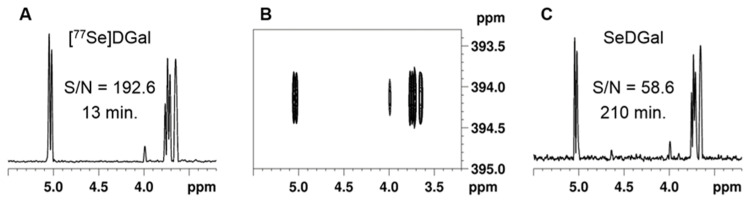
Comparison of signal-to-noise (S/N) ratios of 2D ^1^H-^77^Se CPMG-HSQMBC spectra obtained on [^77^Se]DGal and SeDGal samples of 2 mM each. (**A**) ^1^H (*F2*) projection of 2D ^1^H-^77^Se correlation spectrum of [^77^Se]DGal recorded in 13 min, resulting in an S/N = 192.6; (**B**) representative 2D ^1^H-^77^Se correlation spectrum of [^77^Se]DGal; and (**C**) ^1^H (*F2*) projection of 2D ^1^H-^77^Se correlation spectrum of nonenriched SeDGal recorded in 3.5 h, resulting in an S/N = 58.6.

The sensitivity gain using 99% ^77^Se-enriched SeDGal ([^77^Se]DGal) versus the nonenriched specimen (SeDGal) was checked via comparative 2D ^1^H-^77^Se CPMG-HSQMBC experiments. Figure 1 shows two sets of data corresponding to [^77^Se]DGal (A) and SeDGal (C), respectively, in terms of signal to noise (S/N) ratios and times of data acquisition (Acq) for samples of equal concentrations. The overall sensitivity improvement calculated from the experimental data in Figure 1 is 13.21 in terms of unit acquisition time (Equation (1)):

[S/N(A)/S/N(C)] ∗ [(Acq(C)/Acq(A)]^0.5^ = (192.6/58.6) ∗ (210/13)^0.5^ = 13.21 (1)

This figure compares well with the theoretical value of 12.98 based on isotopic abundances [(99.0/7.63)]. Conversion of this ratio into overall NMR experimental times results in a remarkable ~170-fold [(12.98)^2^] reduction in favor of the enriched specimen for samples of equal concentrations.

**Figure 2:**
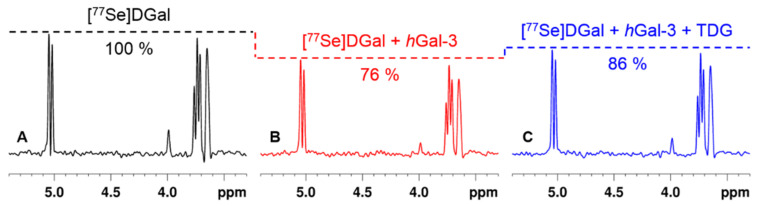
^77^Se-enriched selenodigalactoside ([^77^Se]DGal) binding to *h*Gal-3 and its competitive displacement by thio-di-galactoside (TDG) monitored with the ^1^H-^77^Se CPMG-HSQMBC method. 1D ^1^H (*F2*) cross sections of 2D ^1^H-^77^Se CPMG-HSQMBC experiments (3 min each) performed on samples of (**A**) [^77^Se]DGal (2 mM) in the absence of *h*Gal-3; (**B**) [^77^Se]DGal (2 mM) and *h*Gal-3 (29 μM, i.e., molar ratio = 1:0.0145); and (**C**) [^77^Se]DGal (2 mM), TDG (2 mM), and *h*Gal-3 (29 μM, i.e., molar ratio = 1:1:0.0145). Each spectrum was measured and plotted with identical parameters. At the top of the spectra, dashed lines indicate the overall intensity change of correlation signals of [^77^Se]DGal. In the presence of *h*Gal-3 (**B**), the signal intensity decreased compared with that of free [^77^Se]DGal in (**A**). The addition of TDG causes a rebound of the attenuated [^77^Se]DGal signals (**C**), indicating its competitive displacement by TDG.

To demonstrate the usefulness of ^77^Se-enriched ligand in binding experiments, Figure 2A shows the 1D cross section from a 3-minute 2D ^1^H-^77^Se CPMG-HSQMBC experiment of [^77^Se]DGal (2 mM). This spectrum conveys the reference (100%) signal intensity. It is noteworthy that the S/N ratio is more than adequate in this 3-minute spectrum, leaving room to reduce the concentration of the ligand (and, consequently, that of the protein) in these experiments. The addition of human galectin-3 (*h*Gal-3, 29 μM) to this sample resulted in a decrease in signal intensity (from 100% to 76%, Figure 2B) by line broadening, which indicated binding of the ligand to the protein [25]. The line broadening in Figure 2B was then partially reversed (from 76% to 86%, Figure 2C) with the introduction of thiodigalactoside (TDG, 2 mM) to the sample. This phenomenon is a consequence of reduced relaxation enhancement of [^77^Se]DGal resonances via competition between the two ligands (TDG and SeDGal) for the same recognition site on *h*Gal-3, as explained earlier [25]. It also illustrates that [^77^Se]DGal can be used via its competitive displacement to indirectly monitor the binding of even non-selenated compounds to *h*Gal-3 (or any other target proteins) by our sensitivity-optimized ^1^H-^77^Se CPMG-HSQMBC method.

In sum, we have demonstrated that the enhanced detection sensitivity inherent in our original ^1^H-^77^Se CPMG-HSQMBC sequence receives a further significant boost by using ^77^Se-enriched ligands, such as [^77^Se]DGal, described in this article. The illustrated straightforward introduction of ^77^Se into the glycosidic bond opens wide applicability of ^77^Se-enriched selenoglycosides for biomedically relevant tissue lectins and beyond. In terms of NMR experimental time, this enhancement allows to obtain data in much less time (a factor of about 170 (12.98^2^) between solutions of identical concentrations), and this gain is further documented by the results of a ligand competition experiment. The significant sensitivity gain offered by ^77^Se-enrichment, such as in [^77^Se]DGal, enables, furthermore, a fast and specific detection approach for screening the binding of non-selenated ligands as well to *h*Gal-3. The combined sensitivity advantage of this approach will likely aid NMR to become competitive for binding/screening studies at low concentrations nearing physiological/cellular levels with selenated and non-selenated ligands too. Our present study is a first step toward allowing ^77^Se-enriched selenoglycosides to become versatile tools in interaction analysis by NMR spectroscopy, and inspires new approaches to combine it with synthetic carbohydrate chemistry, as illustrated herein. The current work opens perspectives for using isotopically enriched selenoglycans alone or in combination of ^77^Se with ^2^D, ^13^C, ^15^N, ^19^F, and/or ^31^P for analyzing the structure and dynamics of ligand binding to proteins by taking advantage of different NMR time scales offered by distinct NMR frequencies of the individual isotopes. Therefore, our method has the potential to support drug development through the rapid and detailed investigation of glycan–lectin interactions.

## Data Availability

All data can be directly obtained by contacting the authors.

## Supplementary Materials

The following are available online at https://www.mdpi.com/article/10.3390/pharmaceutics14010201/s1, Figure S1: 500 MHz ^1^H NMR spectrum of di(2,3,4,6-tetra-*O*-acetyl-β-D-galactopyranosyl)selenide (**2**) (99% ^77^Se-enriched, bottom), compared with the spectrum of the same compound with ^77^Se in natural abundance (top) in CDCl_3_; Figure S2: 500 MHz ^1^H NMR spectrum of [^77^Se]DG, **3** (99% ^77^Se-enriched, bottom), compared with the spectrum of SeDG (^77^Se in natural abundance, top) in D_2_O; Figure S3: 125 MHz ^13^C NMR spectrum of [^77^Se]DG, **3** (99% ^77^Se-enriched, bottom), compared with the spectrum of SeDG (^77^Se in natural abundance, top) in DMSO-*d_6_*; Figure S4: 400 MHz ^1^H NMR spectra obtained on samples of [^77^Se]DGal (2 mM) in the absence of *h*Gal-3 (bottom, blue), [^77^Se]DGal (2 mM), and *h*Gal-3 (29 μM, i.e., molar ratio = 1:0.0145) (middle, red), as well as [^77^Se]DGal (2 mM), TDG (2 mM), and *h*Gal-3 (29 μM, i.e., molar ratio = 1:1:0.0145) (top, green), in D_2_O.
