# Supplementary material for: 77Se-Enriched Selenoglycoside Enables Significant Enhancement in NMR Spectroscopic Monitoring of Glycan–Protein Interactions
Source: Pharmaceutics. 2022 Jan 15;14(1):201. doi: 10.3390/pharmaceutics14010201 (PMC8779653; doi:10.3390/pharmaceutics14010201)
Supplement: Supplementary file 1 [file pharmaceutics-14-00201-s001.zip › pharmaceutics-1535854-supplementary.pdf]

pharmaceutics

Supplementary Materials: ^{77}Se-Enriched Selenoglycoside Enables Significant Enhancement in NMR Spectroscopic Monitoring of Glycan–Protein Interactions

István Timári , Sára Balla , Krisztina Fehér , Katalin E. Kövér , and László Szilágyi

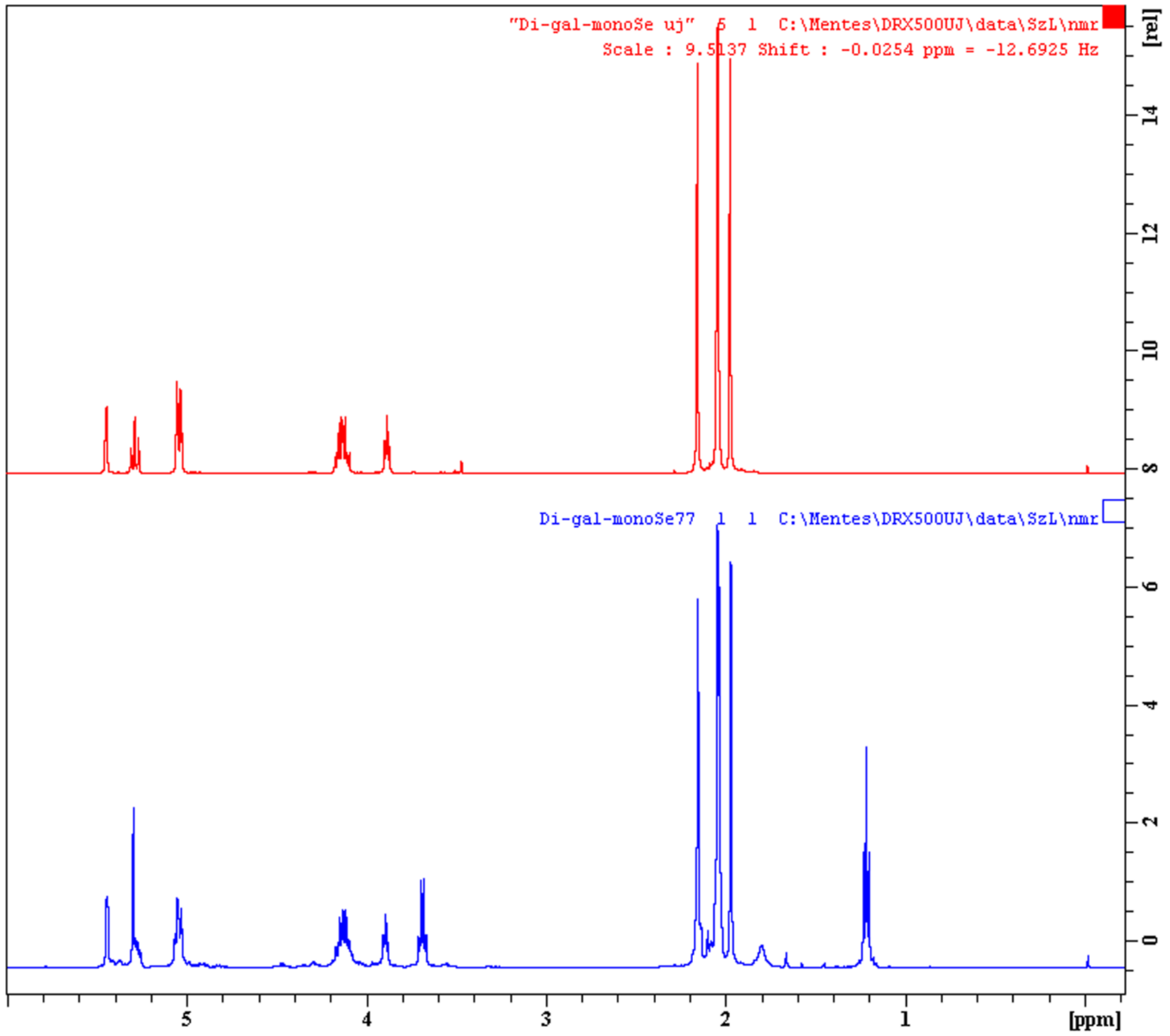

Figure S1. 500 MHz ^{1}H NMR spectrum of di(2,3,4,6-tetra-*O*-acetyl-β-D-galactopyranosyl)selenide (**2**) (99% ^{77}Se-enriched, bottom), compared to the spectrum of the same compound with ^{77}Se in natural abundance (top) in $CDCl_3$.

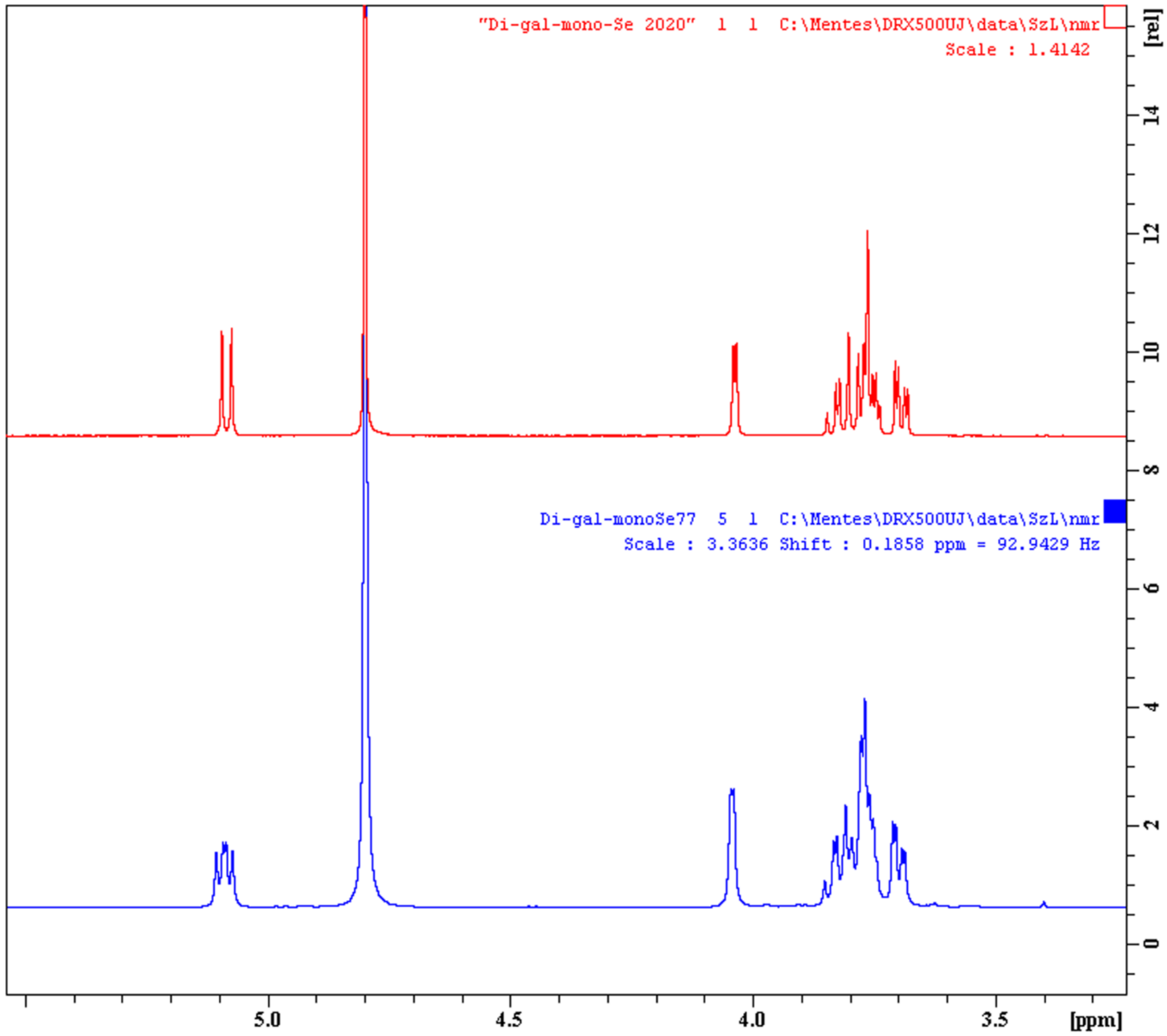

Figure S2. 500 MHz ^{1}H NMR spectrum of [^{77}Se]DG, **3** (99% ^{77}Se-enriched, bottom), compared to the spectrum of SeDG (^{77}Se in natu ral abundance, top) in D_2O.

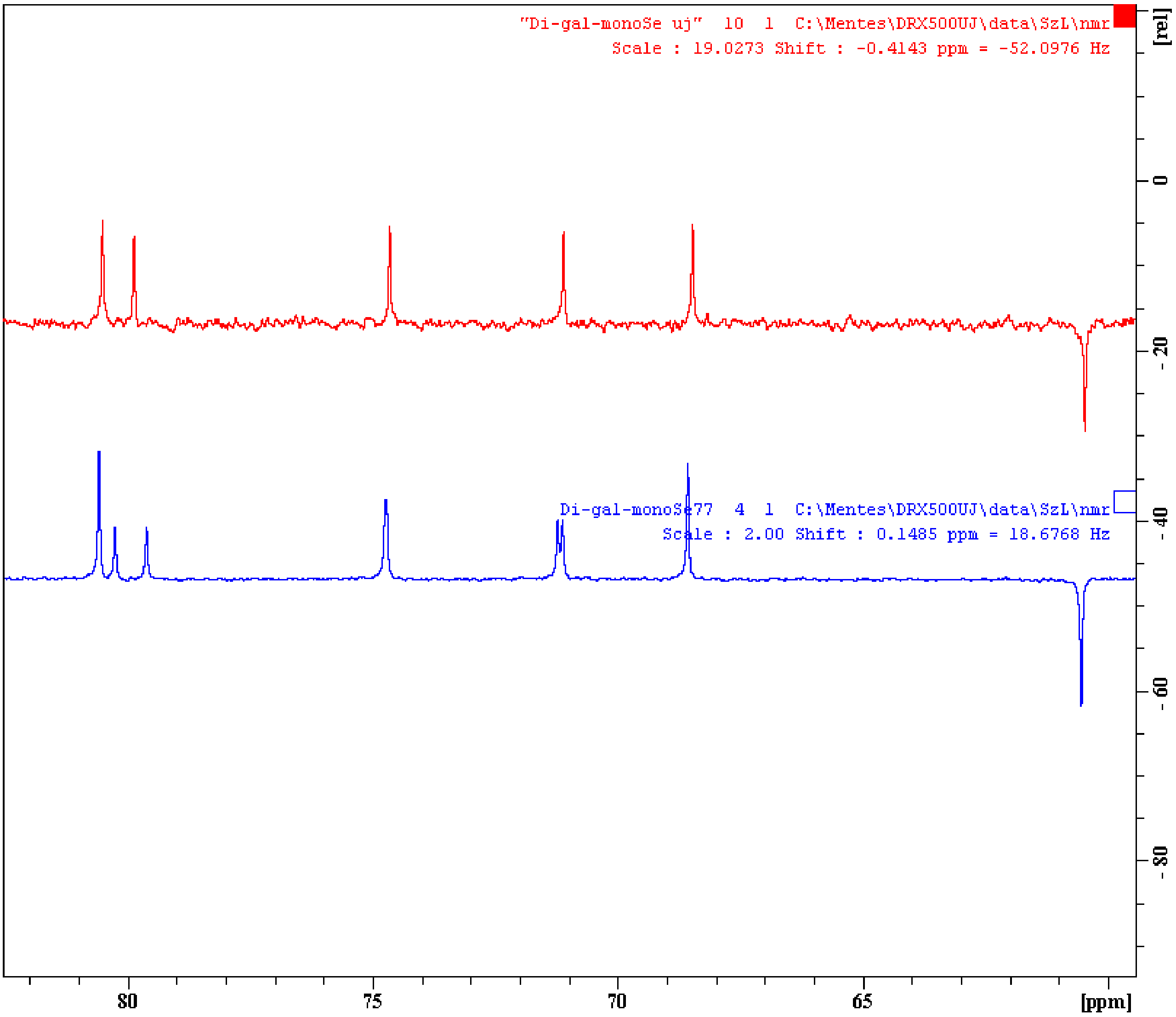

Figure S3. 125 MHz ^{13}C NMR spectrum of [^{77}Se]DG, **3** (99% ^{77}Se-enriched, bottom), compared to the spectrum of SeDG (^{77}Se in natural abundance, top) in DMSO-*d*$_6$.

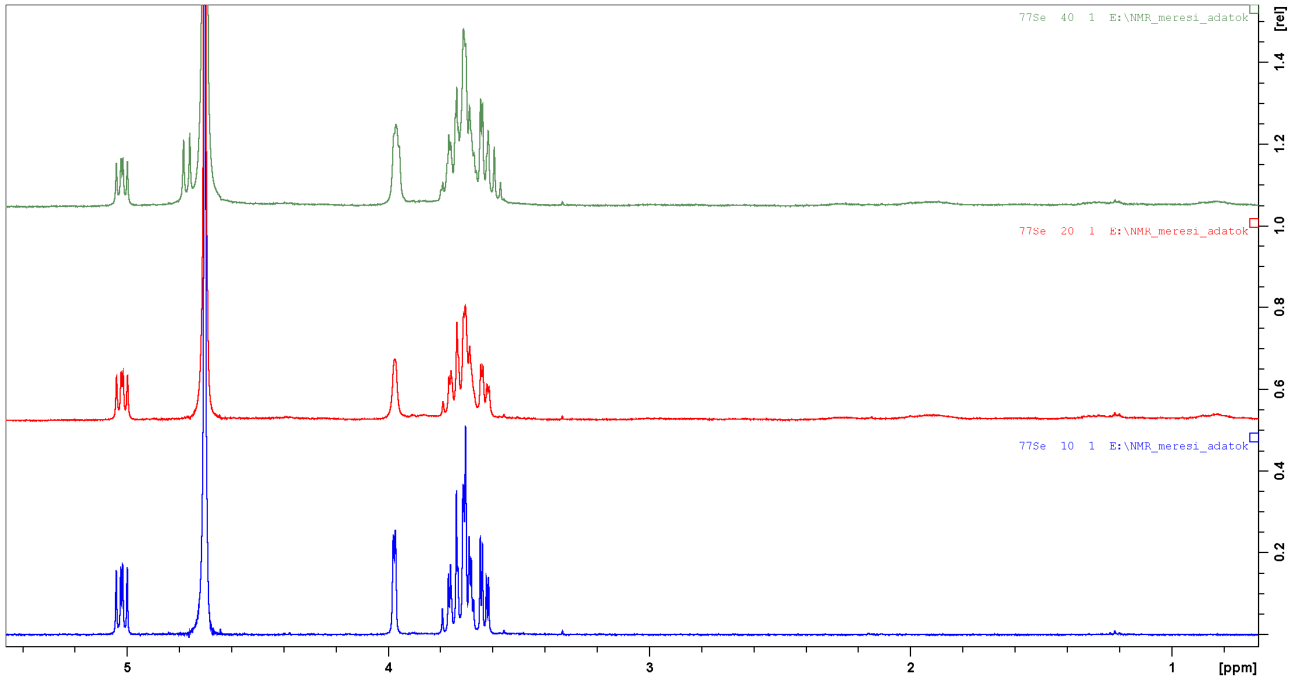

Figure S4. 400 MHz ^{1}H NMR spectra obtained on samples of [^{77}Se]DGal (2 mM) in the absence of *h*Gal-3 (bottom, blue), [^{77}Se]DGal (2 mM) and *h*Gal-3 (29 μM, i.e. molar ratio = 1 : 0.0145) (middle, red) and [^{77}Se]DGal (2 mM), TDG (2 mM) and *h*Gal-3 (29 μM, i.e. molar ratio = 1 : 1 : 0.0145) (top, green) in D_2O.
